# Co-Exposure to Glyphosate and Polyethylene Microplastic Affects Their Toxicity to *Chlorella vulgaris*: Implications for Algal Health and Aquatic Risk

**DOI:** 10.3390/molecules30193972

**Published:** 2025-10-03

**Authors:** Magdalena Podbielska, Małgorzata Kus-Liśkiewicz, Dariusz Płoch, Ewa Szpyrka

**Affiliations:** 1 Faculty of Biotechnology, Collegium Medicum, University of Rzeszów, Pigonia 1 St, 35-310 Rzeszow, Poland; mkus@ur.edu.pl (M.K.-L.); eszpyrka@ur.edu.pl (E.S.); 2 Institute of Materials Engineering, Faculty of Exact and Technical Sciences, University of Rzeszow, Pigonia 1 St, 35-310 Rzeszow, Poland; dploch@ur.edu.pl

**Keywords:** polyethylene microplastics, glyphosate, *Chlorella vulgaris*, combined toxicity, oxidative stress

## Abstract

Polyethylene microplastics (PE-MPs) and glyphosate (GLY) are widespread aquatic contaminants, but their combined effects on microalgae remain poorly understood. This study assessed the individual and joint toxicity of GLY and PE-MPs to the model microalga *Chlorella vulgaris*. Acute (3-day) and chronic (7-day) exposures were performed using GLY at 1–40 mg/L, alone or combined with PE-MPs (10 mg/L). A four-parameter log-logistic (4PL) model was applied to estimate median effect concentrations (EC_50_). After 72 h, the EC_50_ values were 9.77 mg/L for the GLY single system and 2.31 mg/L for the GLY-PE combined system, confirming enhanced toxicity in combined exposures. Co-exposure reduced pigment levels (chlorophyll a, chlorophyll b, and carotenoids) by up to 65% and significantly increased oxidative stress markers, including reactive oxygen species production and malondialdehyde accumulation, compared with single treatments. Antioxidant enzymes (superoxide dismutase and catalase) showed concentration- and time-dependent responses, indicating activation of cellular defense mechanisms. Scanning Electron Microscopy revealed PE-induced aggregation and structural damage to algal cells, particularly at higher GLY concentrations. These findings demonstrate that PE-MPs can amplify the toxic effects of GLY on microalgae and highlight the need for further studies at environmentally relevant concentrations and with different polymer types.

## 1. Introduction

Plastics are produced globally in massive quantities and have become a major source of environmental contamination. Due to their durability and slow degradation, larger plastic debris undergoes fragmentation into microplastics (MPs, <5 mm) and nanoplastics (NPs, <1 µm) [1,2]. These particles are now detected across diverse aquatic systems, where they may interact with other pollutants and represent an emerging ecological threat [3]. PE, one of the most widely produced polymers, is of special concern because of its high abundance in waste streams and its persistence in aquatic environments [4]. These particles accumulate in terrestrial, freshwater, and marine ecosystems, posing ecological risks by disrupting food webs, altering habitats, and serving as carriers for toxic pollutants and microorganisms [5,6]. Research in this field therefore aims not only to elucidate the mechanisms of PE-MPs formation, transport, and toxicity, but also to provide a scientific basis for risk assessment, environmental regulation, and the development of innovative strategies for pollution mitigation. By advancing knowledge in this domain, such studies contribute to global efforts toward safeguarding ecosystem integrity, protecting public health, and fostering sustainable materials management.

Glyphosate (GLY) (IUPAC name: 2-[(phosphonomethyl)amino]acetic acid) is a non-selective, common, and effective herbicide belonging to the organophosphate group. It is one of the most popular herbicides used worldwide. Its mode of action is based on the inhibition of 5-enolpyruvylshikimate-3-phosphate synthase [7]. GLY is highly soluble in water, relatively volatile, and not persistent in soil. Glyphosate has often been detected in surface freshwater (aqueous solution), suspended matter, soil, sediment, groundwater, and even drinking water [8]. It may enter aquatic systems through surface water, by leaching from water and soil, as a spray drift, or as run-off from agricultural applications [9]. The introduction of GLY into the aquatic environment is uncommon. However, GLY is commonly used for the elimination of aquatic weeds in water body margins; thus, leaching and surface runoffs are a potential source of contamination of the aquatic ecosystem. Studies indicate that glyphosate might be toxic to organisms of freshwater biota (both target and non-target aquatic organisms) such as bacteria, microalgae, fish, and amphibians, and is considered an emerging pollutant [10]. Glyphosate exerts toxic effects on *Chlorella* cells by disrupting photosynthesis and oxidative balance. It inhibits the shikimate pathway, reducing the synthesis of essential aromatic amino acids, which impairs protein and pigment production. Exposure leads to reduced growth, chlorophyll degradation, oxidative stress, and structural damage to cell membrane fluidity and permeability by increasing saturated fatty acids with a concomitant decrease in monosaturated fatty acids and phospholipids, ultimately compromising cell viability and metabolic activity [11,12,13,14].

In the aquatic environment, microalgae are a basic primary producer. They play an essential role in maintaining the ecological balance in the ecosystem by producing oxygen and taking part in the global carbon cycle, and they form an integral part of the marine food chain. Microalgae are unicellular and easily cultivated in different trophic modes [15]. They can grow in autotrophic, heterotrophic, or mixotrophic conditions [16]. *Chlorella (C.) vulgaris* is a unicellular microalga with an ellipsoidal cell of 2–10 μm in diameter [17]. It belongs to the green microalgae (*Chlorophyta* family) and has many similar structural elements to plants. *C. vulgaris* are microscopic eukaryotic organisms capable of releasing oxygen through photosynthesis and generating biomass for feed, bioactive compounds, and fuel [18]. The growth cycle of *Chlorella* involves a rapid exponential phase where the microalgae multiply quickly, followed by a stationary phase where their growth is stabilized. Microalgae are sensitive to pollutants present in the aquatic environment, including MPs/NPs. The impact of MPs on microalgae is of great importance in environmental research, including the assessment of the effects of MP/NP pollution on the aquatic environment. Recent studies showed that MPs/NPs affected microalgae, causing structural damage, growth inhibition, reduced photosynthetic capacity, and oxidative stress [19,20]. The potential toxicity of MPs/NPs to microalgae increases with a decrease in the particle size. Moreover, small-sized plastics present a high sorption capacity for other chemicals (organic pollutants and heavy metals), and as a consequence modify the bioavailability of these chemicals. The current knowledge on pesticide adsorption by MPs/NPs is limited; their adsorption rate by polyethylene MPs (PE-MPs) can be correlated with pesticide hydrophobicity, and this may explain the synergistic effect of PE-MPs with a variety of pesticides [21]. Previous studies indicated a potential general synergism, with MPs/NPs intensifying the toxic effects of pesticides. Felten et al. (2020) reported a decrease in the number and quality of broods, a delayed first brood, reduced fertility, and an increase in the mortality of *Daphnia magna* exposed to deltamethrin combined with PE-MPs [22]. A study in *Cyprinus carpio* confirmed that the presence of PE-MPs and glyphosate affected its swimming speed, acceleration, and social behavior. This combination altered the metabolome and microbiome and destroyed the intestinal barrier in common carp [9]. Yu et al. (2021) indicated that PS-MPs and glyphosate activated plant antioxidant defense systems by increasing the activity of antioxidative enzymes, to cope with oxidative stress [23]. The authors observed synergistic effects when *Salvinia cucullata* was exposed to high concentrations of PS-MPs (≥15 mg/L) and glyphosate at the concentration of 25 mg/L. PS-NPs increased the malformation rate of *Danio rerio* when exposed to 1,1-Dichloro-2,2-bis (4-Chlorophenyl) ethylene [24]. However, Garrido et al. (2019) demonstrated that *Isochrysis galbana* appeared to better tolerate the combined effects of chlorpyrifos and PE-MPs [25]. Another study showed a potential protective effect against pesticide-induced growth inhibition. In turn, Horton et al. (2018) reported no interaction between deltamethrin and PS-MPs in *D. magna* [26].

Quantitative assessment of MP toxicity is fundamentally rooted in elucidating concentration–response relationships. Among the array of modeling frameworks available in ecotoxicological research, the four-parameter log-logistic (4PL) model is widely adopted due to its ability to represent key biological dynamics: namely, the asymptotic responses at low and high concentrations, the steepness of the effect gradient, and the inflection point corresponding to the median effect concentration (EC_50_) [27]. The 4PL model thereby yields a statistically robust and biologically interpretable EC_50_, facilitating meaningful comparisons across diverse species, MP forms, and endpoints; it is a standard choice when symmetric concentration–response behavior is assumed, owing to its relative simplicity and numerical stability [28]. In ecotoxicology, this approach provides a rigorous analytical foundation for evaluating MPs’ impacts across trophic levels and exposure scenarios.

The aim of this study was to investigate the toxicity effects of GLY and co-pollutant GLY-PE on microalgae and dose–response relationships using a nonlinear regression model. To fulfill this knowledge gap for the first time, a 4PL nonlinear regression model was used to estimate the toxicity value—median EC_50_—for contaminants in a single and a combined system. Moreover, additional analyses to confirm the toxicity of GLY in the single system and GLY-PE in the combined system, which included pigment content and oxidative stress parameters, were also performed.

## 2. Results and Discussion

### 2.1. Effects of GLY and GLY-PE on the Toxicity of Microalgae

**Figure 1:**
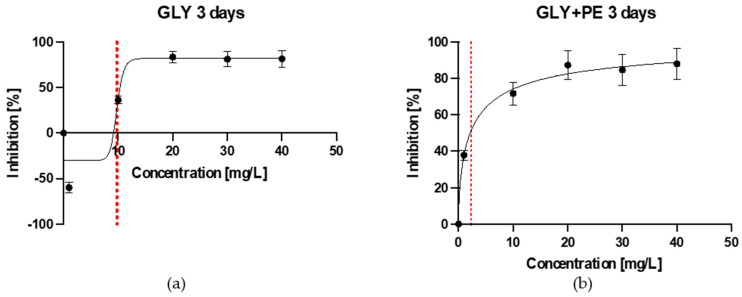
Dose–response curve fitted using the 4-parameter log-logistic (4PL) model for GLY-treated (**a**) and GLY-PE-treated (**b**) *C. vulgaris* microalgae in the concentration range of 1–40 mg/L after 3 days. The red intermittent lines represent the designated EC_50_.

In the present study, the acute toxicity test, performed according to the OECD guidelines (No. 201) [28], showed that after 72 h, the EC_50_ values of GLY and GLY-PE to *C. vulgaris* were 9.77 mg/L and 2.31 mg/L, respectively (Figure 1a,b).

An acute toxicity test represents the initial phase in evaluating the harmful effects of toxic substances and serves as a basis for conducting chronic toxicity assessments. The EC_50_ is a critical measure of toxicology, and refers to the concentration at which 50% of the exposed organisms exhibited an effect, making it a crucial measure of a substance’s toxicity. Typically, a lower EC_50_ value indicates a higher level of toxicity. The primary objective of this test was to determine the EC_50_ for GLY and GLY-PE in test organisms. According to the literature, EC_50_ values vary significantly depending on the type of MP and the organism tested. A few studies have calculated EC_50_ values for MPs and their interactions with contaminants. The 48 h EC_50_ values of 1 μm and 10 μm PS to *D. magna* were 66.97 mg/L and 199.94 mg/L, respectively. When PS-MP was combined with roxithromycin, the toxicity increased, indicating significant interactions between the two pollutants, the EC_50_ value was 20.28 mg/L [29]. The EC_50_ of PMMA-MPs for *P. similis* was 44.0 mg/L, but this value significantly decreased when PMMA-MPs were co-exposed with the metal(loid)s (As and Cu) [30]. The combination of PS nanoplastics and diphenhydramine showed synergistic effects, with increased embryo mortality and malformations in zebrafish embryos compared to individual exposures [31]. The combined PE-MP and lead acetate system showed increased bioaccumulation and synergistic toxicity effects in freshwater grass shrimp, emphasizing the enhanced toxicity when both contaminants are present [32]. Our results confirm that the presence of MPs can alter the toxicity of contaminants, often increasing their harmful effects. Synergistic interactions between MPs and contaminants highlight the need for comprehensive risk assessments considering combined exposures.

### 2.2. Effects of GLY and GLY-PE on Microalgal Chlorophyll Content Alteration by GLY and GLY-PE on the Growth of Microalgae

**Figure 2:**
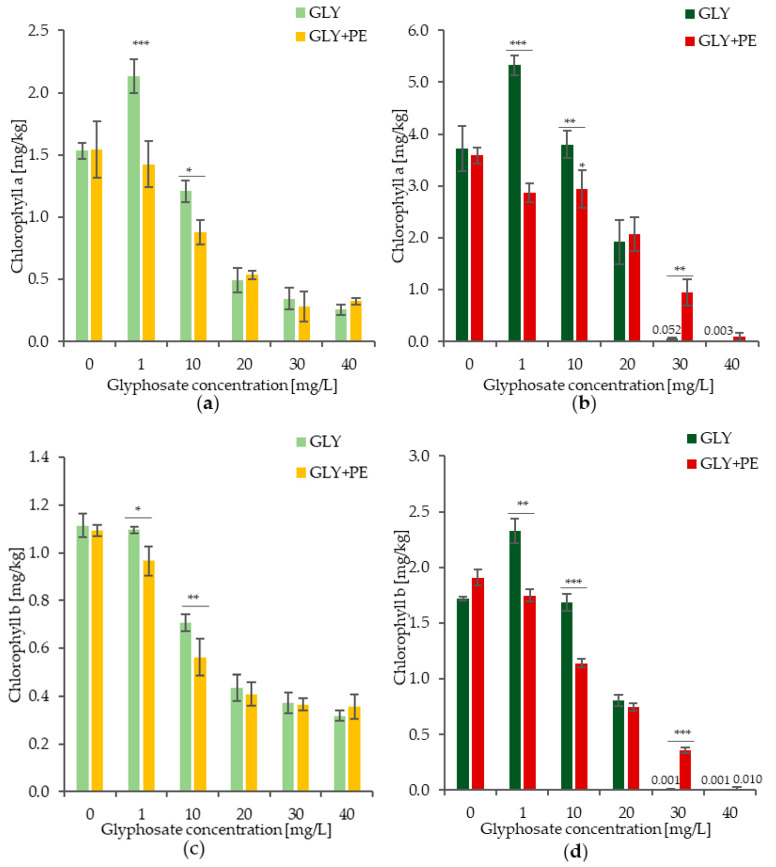

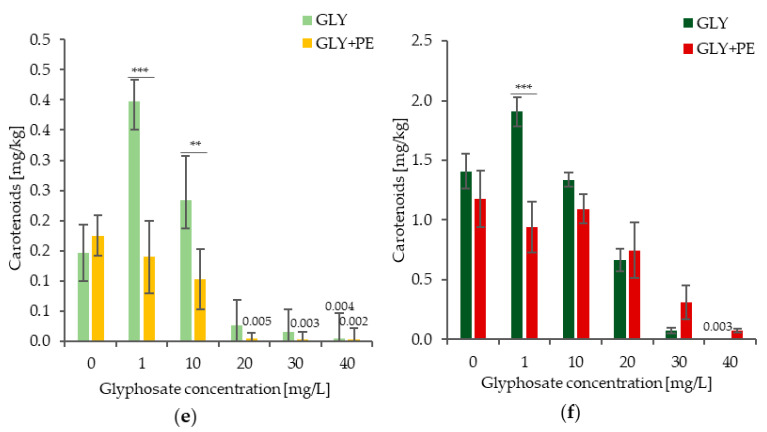
Effects of exposure to the GLY single and GLY-PE combined systems on chlorophyll a after 3 days (**a**) and 7 days (**b**); chlorophyll b after 3 days (**c**) and 7 days (**d**); and carotenoids after 3 (**e**) and 7 days (**f**). The data values represent the mean ± SD (n = 3), and the comparison was between the GLY single system and GLY-PE combined system. The asterisk represents statistical significance; * *p* < 0.05, ** *p* < 0.01, *** *p* < 0.0001.

The changes in the chlorophyll a, chlorophyll b, and carotenoid content, and the effect of the GLY single system at five concentrations and the GLY-PE combined system on *C. vulgaris*, are shown in Figure 2a–f. The use of the GLY-PE combined system resulted in a reduction in the pigment production for GLY concentrations of 1–20 mg/L. After 3 days, for the GLY-PE combined system at concentrations of 1 and 10 mg/mL, significant reductions were observed in the levels of chlorophyll a, by 33.2% and 27.1%, respectively, (Figure 2a); chlorophyll b, by 11.8% and 20.5%, respectively, (Figure 2c); and carotenoids, by 65.1% and 56.0%, respectively, compared to the GLY single system (Figure 2e). After 7 days, a decrease in the pigment concentration was observed following the treatment with the GLY-PE combined system at concentrations of 1–20 mg/L. A decrease in GLY-PE concentration to 1 and 10 mg/L, respectively, led to reductions of 46.2% and 22.7% for chlorophyll a, 24.9% and 32.3% for chlorophyll b, and 50.5% and 18.5% for carotenoids (Figure 2b,d,f). These results were statistically significant. At the concentration of 30 mg/L, pigment inhibition was alleviated after 7 days of the experiment, and higher pigment production was observed in the samples exposed to GLY-PE.

Pigment content has been used as a sensitive biomarker for toxicity effects induced in microalgae by various aquatic xenobiotics [13]. In the present experiments, with an increase in the GLY concentration, both when used separately and as a part of the GLY-PE combined system, the pigment content of *C. vulgaris* decreased. However, an interesting phenomenon was the transient increase in pigments observed at low GLY concentrations (1 mg/L). This can be interpreted as a hormesis effect—mild chemical stress induced by a low dose of herbicide activates microalgae’s defense mechanisms and may temporarily stimulate their metabolism. This phenomenon is consistent with previous reports indicating that low doses of pesticides can induce an adaptive response in microalgae, whereas higher doses lead to growth inhibition and oxidative stress [20]. The decrease in pigment content indicates that the photosynthetic system of microalgae was inhibited. We observed that these effects were time-dependent. The inhibitory effect of the GLY-PE combined system gradually increased with prolonged exposure time. This decrease implies that the light reaction was inhibited, which could have resulted from the shading effect of the MPs, forming heteroaggregates with microalgae. This effect could hinder light and nutrient penetration into cells [14]. The negative effect of MPs on photosynthetic pigments can also be explained by the accumulation of intracellular reactive oxygen species (ROS), which can damage cell structure and block chlorophyll synthesis [11]. The results obtained are consistent with those of other similar studies, such as that conducted by Yang et al. (2023) [33]. They stated that the addition of 50 μm PE-MPs affected the growth of the two microalgae *Nitzschia closterium f. minutissima* and *P. donghaiense*, resulting in the inhibition of Fv/Fm, where the inhibitory effect of PE-MPs was more significant in the case of *P. donghaiense*. LDPE-MPs contributed to inhibition rates by 85% in the case of the chlorophyll content [33]. The results of Wang et al. (2023) showed that after exposure of *C. vulgaris* to PE-MPs at different concentrations and of different particle sizes for 11 days, pigment contents were not affected [34]. The results of other studies showed that both PVC and PP negatively affected chlorophyll a concentration in *C. pyrenoidosa* and *Microcystis flos-aquae*. In their case, when the PVC concentration exceeded 250 mg/L, the chlorophyll a content of *C. pyrenoidosa* was reduced by 55.23% versus the control group [35]. Z. Li et al. (2022) showed that the presence of PS-MPs and sulfadiazine resulted in a significant reduction in chlorophyll a, b, and c levels in microalgal cells [36]. W. Yang et al. (2021) found that the pigment content in microalgae was significantly reduced after 48 h of exposure to PS [37]. Zhao et al. (2019) showed that 1 µm of PVC at the concentration of 100 mg/L reduced the chlorophyll a level in *Karenia mikimoti* [38].

### 2.3. Morphological Properties

**Figure 3:**
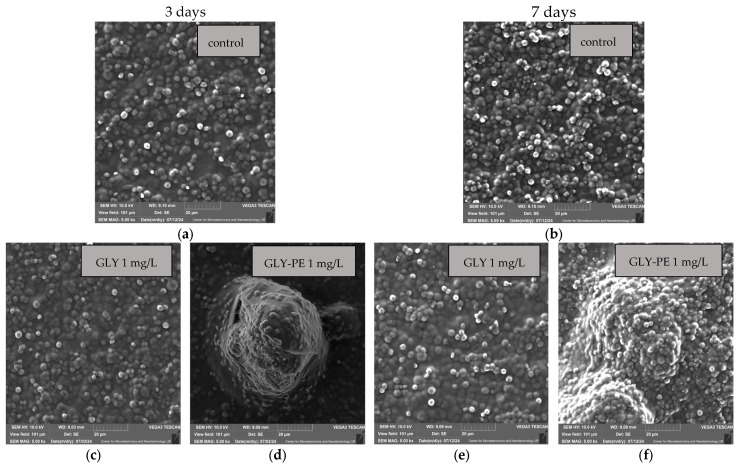

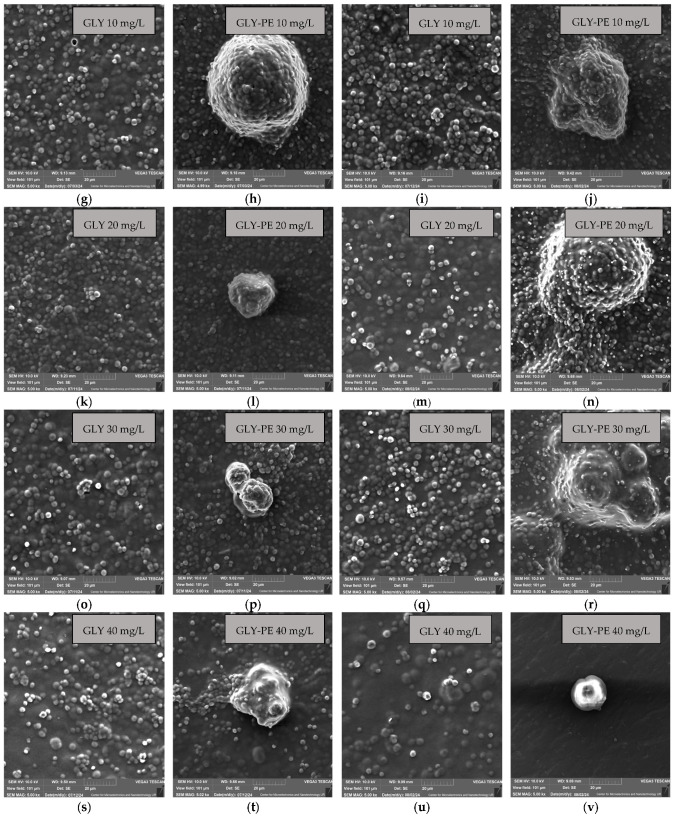
SEM images of the GLY single system and the GLY-PE combined system of exposure after 3 days (first column) in the control (**a**) and in the samples at GLY and GLY-PE concentrations of 1 mg/L (**c**,**d**); 10 mg/L (**g**,**h**); 20 mg/L (**k**,**l**); 30 mg/L (**o**,**p**); and 40 mg/L (**s**,**t**), respectively, and after 7 days (second column) in the control (**b**) and in the samples at GLY and GLY-PE concentrations of 1 mg/L (**e**,**f**); 10 mg/L (**i**,**j**); 20 mg/L (**m**,**n**); 30 mg/L (**q**,**r**); and 40 mg/L (**u**,**v**), respectively.

**Figure A1:**
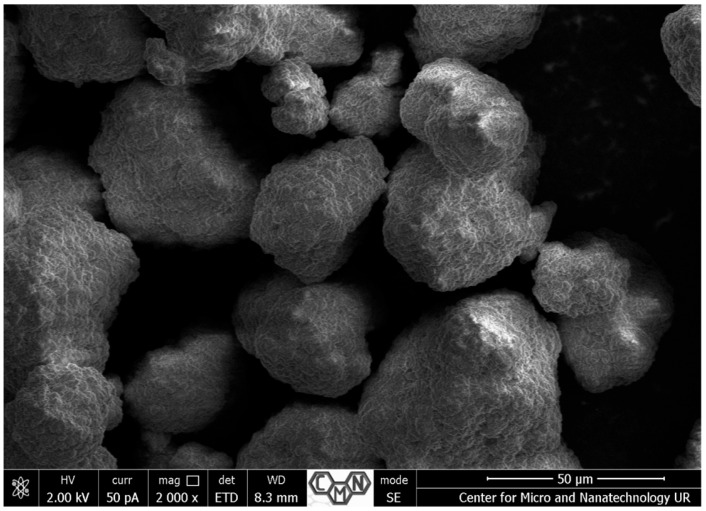
SEM image of the PE-MPs.

As shown in Figure A1, Appendix A, the PE-MPs were of uneven size and irregular shapes. The SEM images of *C. vulgaris* exposed to the GLY single and GLY-PE combined systems are presented in Figure 3a–v. The Scanning Electron Microscopy (SEM) observations confirmed that in the presence of PE, *C. vulgaris* cells formed heteroaggregates (with dimensions in the range of 8.61–19.18 µm), often depositing on the particle surface. This interaction promoted cell wall deformation and sometimes membrane disruption. These effects were particularly pronounced at higher concentrations of GLY combined with PE, indicating cumulative physical and chemical stress. Images at 40 mg/L GLY-PE revealed that all cells were damaged, confirming the synergistic nature of the toxicity and correlating well with the biochemical results (increased ROS and malondialdehyde (MDA)). Wang et al. (2016) confirm that heteroaggregations can cause physical damage to algae, such as changes in the structure of the cell wall [39]. Damage to the cell membrane was also observed in the present study. This can affect nutrient and energy transport and reduce cytomembrane fluidity, thus inhibiting microalgae growth.

### 2.4. Effects of GLY and GLY-PE on SOD and CAT Activities and MDA and ROS Levels

**Figure 4:**
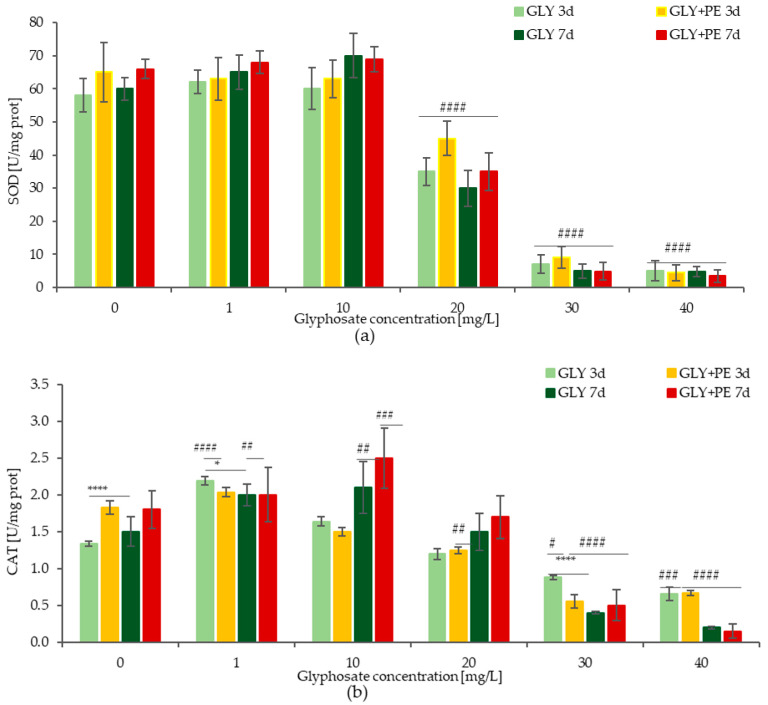

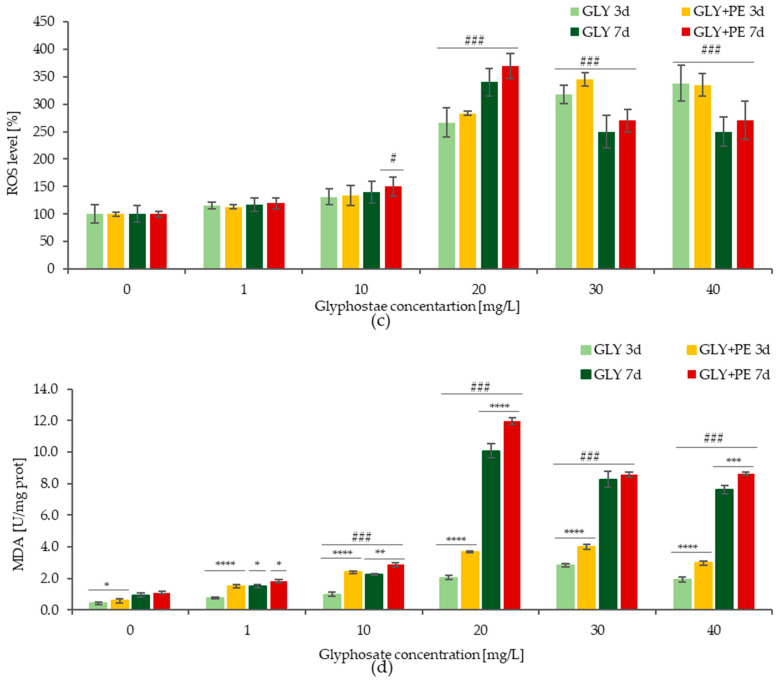
Variations in the SOD activity (**a**), CAT activity (**b**), ROS level (**c**), and MDA content (**d**) of *C. vulgaris* in response to different concentrations of the GLY single system and the GLY-PE combined system. Data values represent the mean ± SD (n = 3). Significance denoted as ^#^ *p* < 0.05, ^##^ *p* < 0.01, ^###^ *p* < 0.001, and ^####^ *p* < 0.0001 is given for samples treated with GLY and GLY-PE compared to the control, while significance denoted as * *p* < 0.05, ** *p* < 0.01, *** *p* < 0.001, and **** *p* < 0.0001 is given for the GLY single system and the GLY-PE combined system.

Figure 4a illustrates the changes in the superoxide dismutase (SOD) activity in microalgae after their exposure to the GLY single and GLY-PE combined systems for 3 and 7 days. After 3 days of exposure, the SOD activity in the GLY single system showed slight increases at GLY concentrations of 1 mg/L and 10 mg/L, amounting to 6.9 and 3.4%, respectively. At concentrations of 20, 30, and 40 mg/L, decreases in SOD activity of 39.7%, 91.4%, and 91.9%, respectively, were observed when compared to the control sample. The SOD activity in the GLY-PE combined system with GLY concentrations of 1 mg/L and 10 mg/L was almost at the same level, and a decrease was observed in the concentration range of 20–40 mg/L, amounting to 30.8–93.1% versus the control samples. When the GLY single system was compared to the GLY-PE combined system, an increase in SOD activity was observed after the GLY-PE treatment. After 7 days of exposure to the GLY single system at concentrations of 1 mg/L and 10 mg/L, increases in the SOD activity were observed, amounting to 8.3% and 16.7%, respectively. In the concentration range of 20–40 mg/L, SOD activity decreased by values in the range of 50–92.2% when compared to the control samples. For the GLY-PE combined system at concentrations of 1 and 10 mg/L, SOD activity increased by approx. 3% and 9%, respectively. At concentrations of 20, 30, and 40 mg/L, SOD activity decreased by 28.6%, 86.4%, and 92.8%, respectively, versus the control samples. The comparison of the GLY single system to the GLY-PE combined system after 7 days revealed that SOD activity increased after treatment with the GLY-PE combined system at concentrations of 1 and 20 mg/L by 4.6% and 16.5%, respectively.

Figure 4b presents the changes in catalase (CAT) activity. In the present study, 3 days after treatment with GLY at concentrations of 1 mg/L and 10 mg/L, CAT activity increased by 64.2% and 22.4%, respectively, when compared to the control samples. For concentrations within the range of 20–40 mg/L, CAT activity decreased by values within the range of 34.3–50.7% versus the control samples. After treatment with the GLY-PE combined system at the concentration of 1 mg/L, CAT activity increased by 10.9% when compared to the control samples. In the concentration range of 10–40 mg/L, CAT activity decreased by values in the range of 18.5–69.6% compared to the control samples. Taking into account the data after GLY single system and GLY-PE combined system treatments, a statistical decrease in CAT activity of 7.3–63.6% was observed during the 3 days of the experiment. In turn, after 7 days of the experiment, an increase of 19% was observed for the concentration of 10 mg/L.

Figure 4c presents the changes in ROS level after 3 and 7 days of the experiment. In the present study, at concentrations of 1 and 10 mg/L, both in the GLY single system and the GLY-PE combined system, the changes in ROS level did not exceed 50% compared to the control samples after 3 and 7 days. For concentrations of 20, 30, and 40 mg/L, the changes in ROS level were statistically significant after 3 days of treatment for the GLY single system. The increases in ROS level were 166%, 218.2%, and 238.2%, and for the GLY-PE combined system, the increases were 183.2%, 244.8%, and 335.2%, respectively, compared to the control samples. After 7 days at the concentration of 20 mg/L, the increases were at the highest level, 240% in the GLY single system and 270% in the GLY-PE combined system, compared to the control samples. For concentrations of 30 mg/L and 40 mg/L, the increases in ROS level were alleviated and amounted to 150% and 170%, respectively. Differences in ROS levels between microalgae treated with the GLY single system and the GLY-PE combined system did not exceed 20%.

The changes in the MDA level in response to exposure to the GLY single and GLY-PE combined systems at different concentrations are shown in Figure 4d. After 3 days of exposure, the MDA content in the microalgal cells responded positively to GLY and the GLY-PE at each treatment concentration. After 3 days of exposure, the MDA content gradually increased for GLY concentrations in the range of 1–30 mg/L, especially after treatment with the GLY-PE combined system. An increase in the MDA content of 1.3 to 2.5 times was observed following treatment with the GLY-PE combined system at concentrations of 1, 10, 20, and 30 mg/L when compared to the GLY-treated samples. These results were statistically significant (*p* < 0.001). After 7 days of exposure, the highest increase in the MDA content was observed after treatment with GLY-PE at the concentration of 20 mg/L. The increase in MDA after treatment with GLY-PE was significantly higher, by 19% (*p* < 0.0001), than after treatment with GLY alone. At concentrations of 30 and 40 mg/L, the increase in the MDA content was alleviated and amounted to a protein reduction of 7.6–8.6 U/mg.

The presence of xenobiotics in the environment is unfavorable for microalgae, causes changes in microalgae growth, and has adverse effects on the enzymatic activity in microalgal cells [40]. Factors such as organic contaminants, heavy metals, organic acids, and salt are the main abiotic stressors. To cope with them, microalgae have developed various mechanisms involving different strategies to maintain cellular homeostasis and prevent damage to cellular components. One of these strategies is the production of enzymatic antioxidants to prevent the harmful effects of ROS generated within plant cells and maintain them under oxidative stress [41]. Excess ROS can damage the cell membrane system of the organism and eventually inhibit its growth. Antioxidative enzymes, such as SOD, CAT, peroxidase, and ascorbate peroxidase, play a pivotal role in minimizing the oxidative damage caused by ROS. These enzymes eliminate various types of ROS and convert them into less reactive compounds, thus reducing their harmful effects [42]. Being the first enzyme that directly eliminates ROS, SOD is considered to be the first barrier in the intracellular antioxidant system and is a potentially effective marker of early oxidative damage induced by MPs and xenobiotics [43]. This enzyme can transform radical superoxide into H_2_O_2_. SOD catalyzes the O_2_ dismutation to molecular oxygen and H_2_O_2_, which is subsequently removed by CAT, an important antioxidant enzyme that can convert significant amounts of H_2_O_2_ generated in peroxisomes into H_2_O. The enhancement in CAT activity is considered to be an adaptive characteristic preventing antioxidative stress damage, suggesting that MPs induce excessive ROS production and cause damage to the oxidative system of microalgae. MDA serves as a marker for lipid peroxidation. It is formed when polyunsaturated fatty acids are attacked by oxygen-derived free radicals, resulting in the formation of lipid hydroperoxides [44].

This research shows that low concentrations of the GLY single and the GLY-PE combined systems did not affect the production of ROS and the accumulation of MDA. However, a high concentration and the presence of PE significantly increased ROS production; this effect was visible on the third day of the experiment. On day 7, the ROS activity decreased at concentrations of 30 and 40 mg/L; however, it should be taken into account that the cell count in the samples was much lower. The accumulation of ROS can also cause oxidative damage to cells and lipid peroxidation. Membrane lipid peroxidation can affect the fluidity of the membrane system, which also affects the microalgal photosynthetic system located in the thylakoid membrane. Damage to the microalgal photosynthetic system was confirmed in these studies. After treating the samples with GLY at low glyphosate concentrations, the SOD activity increased significantly, but as the glyphosate concentration increased, the SOD activity gradually decreased. SOD is the main ROS-scavenging enzyme in *C. vulgaris* cells; therefore, when its activity is induced after treatment with low concentrations of glyphosate, it can rapidly quench ROS. At high concentrations of GLY and PE, the activity of SOD was reduced, so ROS could not be removed quickly. The presence of PE increased the amount of ROS produced, which influenced the activity of SOD. Thus, SOD plays an essential role in protecting *C. vulgaris* cells against ROS, especially at low concentrations of glyphosate.

In the literature, evidence for the influence of both MPs and pesticides on microalgae antioxidant enzymes has been provided, highlighting their individual and combined effects on the microalgae growth and the antioxidant enzyme activity. Iummato et al. (2019) [45] studied the effects of a commercial glyphosate formulation at concentrations of 0, 4, 6, and 8 mg/L on the parameters of oxidative stress in the green phytoplanktonic microalga *Scenedesmus vacuolatus*. The parameters of oxidative stress were significantly affected, showing an increase in ROS. They also observed oxidative damage to lipids and proteins, and decreased activity of the antioxidant enzymes SOD and CAT. Exposure to low-density PE-MPs (<5 μm) over a 20-day period at concentrations of 10 mg/L and 100 mg/L resulted in oxidative stress, leading to lipid peroxidation [46]. Li et al. (2022) [47] showed that in addition to PE, 0.3 mg/L sulfamethoxazole significantly decreased toxicity in the combined system due to the “shelter” effect of MP absorption. In this study, the MDA content in the PE combined system was higher than that in the sulfamethoxazole single system, and it simultaneously showed lower SOD activity compared to treatment with sulfamethoxazole alone. The authors also demonstrated that PS and sulfamethoxazole in the combined system were highly affected by the activity of SOD, MDA, and ROS. They indicated that cytomembrane damage was one factor that contributed to combined system toxicity. A study by Senousy et al. (2023) indicated that total protein content, and SOD, CAT, and peroxidase activity, increased significantly in the presence of 25 mg/L of low-density PE-MP, by 1.37, 3.52, 2.75, and 1.84 times versus the controls, and also showed adverse effects of LDPE-MPs on the marine microalga *Chaetoceros calcitrans* [48].

The observed synergistic effect of combining GLY and PE-MPs can be explained by several mechanisms. First, MPs are characterized by a high specific surface area and the ability to adsorb organic compounds, which could influence the bioavailability of GLY for *C. vulgaris* cells. Second, the presence of PE-MPs could lead to a “shadowing” effect, limiting light penetration into the cells and further inhibiting photosynthesis. Third, surface interactions between PE-MPs and cells promote the formation of heteroaggregates, which could hinder gas exchange and nutrient access. All of these factors can exacerbate oxidative stress, as evidenced by the observed increase in ROS levels and increased lipid peroxidation, which we confirmed in our research.

At the same time, the limitations of our study should be emphasized. We focused on a single type of PE-MPs, with a single size fraction (34–50 μm), and on relatively high concentrations of GLY and PE-MPs compared to environmental levels. While this range captured a clear toxic effect and allowed for the estimation of EC_50_ values, it makes it difficult to directly relate the results to natural conditions. Therefore, future studies should consider a broader range of polymers and particle sizes, and lower environmental contaminant concentrations, to better reflect realistic exposure scenarios in aquatic ecosystems.

## 3. Materials and Methods

### 3.1. Chemicals and Materials

PE white powder (CAS: 9002-88-4) with a particle size of 34–50 μm was purchased from Sigma Aldrich (St. Louis, MO, USA). The stock solution of 1000 mg/L PE was prepared by dispersing dry powder in ultrapure water (water purification system SolPure XiO, Elkar, Toruń, Poland) with sonification for 30 min, and then 25 mg TWEEN 20 (BioShop, Burlington, ON, Canada) was added. The GLY analytical standard was purchased from HPC Standards GmbH (Cunnersdorf, Germany). A stock solution of 1000 mg/L of GLY was prepared by dissolving the standard in ultrapure water. The prepared PE and the GLY solutions were stored in a refrigerator at 4 °C.

### 3.2. Microalgae Cultivation

*C. vulgaris* was obtained from CCAP (The Culture Collection of Algae and Protozoa) (Dunbeg, OBAN, Scotland, UK). The strains were activated in a sterile BBM medium (Sigma Aldrich, USA). The microalgae were grown at 25 °C ± 1 °C under blue–red light at a ratio of 1:5 with an intensity of 900 ± 50 lx and a 12 h–12 h light–dark cycle. The microalgae were cultured to be at the logarithmic growth stage for subsequent tests. The initial cell count was 1 × 10^6^ cells/mL.

### 3.3. Toxicity Assay

The toxicity test in the study was performed according to the OECD guidelines (No. 201) [49]. Microalgae exposure experiments (3 days and 7 days) were conducted with five concentrations of GLY (1 mg/L, 10 mg/L, 20 mg/L, 30 mg/L, and 40 mg/L) for the single system, and a mixture of PE at 10 mg/L and GLY at five concentrations for the combined system [17].

Exposure times were selected to represent different types of toxicological effects. Three-day exposure was chosen to represent acute exposure, reflecting the short-term effects of contaminants on microalgal physiology. In contrast, a 7-day duration was chosen to represent chronic exposure, allowing assessment of long-term effects and potential adaptive or cumulative responses. This distinction between short- and long-term exposure is commonly applied in ecotoxicological studies to capture immediate and prolonged impacts of contaminants. The OECD Guidelines suggest using concentrations causing effects between 0% and 100% to allow for dose–response curve fitting. The range should span from no observed effect (NOEC) to a near-complete effect (e.g., 100% mortality or inhibition). For no observed effect, a concentration of 1 mg/L was chosen, then spaced logarithmically by 10 mg/L. A concentration of 20 mg/L was used, on the basis of data provided in the PPDB, as the EC_50_ (19 mg/L). The highest concentration (40 mg/L) was determined in preliminary experiments to cause approximately 100% growth inhibition, and an intermediate value of 30 mg/L was included to capture the transition zone of the dose–response curve. Although these concentrations exceed those typically found in natural aquatic environments, their use allowed accurate modeling of the dose–response relationship and estimation of EC_50_ values [8,49]. The concentration of PE-MPs (10 mg/L) was chosen based on previous ecotoxicological studies with microalgae, where similar levels were applied to ensure reproducibility and comparability across experiments. This value also reflects an experimentally feasible concentration that produces detectable biological responses without causing excessive aggregation or sedimentation of particles [47].

The investigation was carried out in 20 mL of the *C. vulgaris* suspension culture in 50 mL Erlenmeyer flasks [47]. To prevent sedimentation of the cells and particles, the samples were shaken continuously on an orbital shaker at 80 rpm (3001, GFL, Lauda-Königshofen, Germany). Quality control was maintained throughout the experiment. To ensure quality control, all experiments were conducted under strictly sterile conditions. Only glassware was used in the experiment, to avoid potential contamination with other MPs and NPs from lab equipment. Before the test, all glassware in the study was soaked in acid and washed with acetone and ultrapure water and then stored in an oven at 450 °C for 5 h. All microalgae samples were covered with aluminum foil. Cultures were handled under aseptic conditions to prevent microbial contamination. Each treatment was performed in triplicate. When the effect of the GLY single system was tested, the control samples consisted of a pure algae culture without the addition of GLY or PE. When the effect of GLY-PE was tested, the control samples consisted of an algae culture with 10 mg/L PE but without GLY. Continuous orbital shaking was applied to minimize sedimentation of particles and cells during incubation.

The samples were collected after 3 and 7 days, and the cell count was determined using QuadCount (Accuris Instruments, Edison, NJ, USA). The growth inhibition rate (IR) was calculated according to Equation (1).

(1) $$IR\left(\%\right)=(1-\frac{T}{C})\cdot 100\%$$

where T is the number of cells per mL in the treated group, and C is the number of cells per mL in the control group. IR < 0 indicates growth promotion, and IR > 0 indicates growth inhibition.

Dose–response relationships were analyzed using nonlinear regression to model the effect of increasing concentrations of test substances on biological response (e.g., number of surviving individuals). A four-parameter log-logistic (4PL) model was employed.

(2) $${EC}_{50}=D+\frac{a-d}{1+({\frac{x}{c})}^{b}}$$

where EC_50_ D is the dose, a is the lower limit of response, d is the upper limit of response, c is the EC_50_ value (midpoint of the curve), and b is the slope of the curve (indicates sensitivity of the response to dose change).

The model parameters were estimated using nonlinear least squares optimization. The EC_50_ values were derived directly from the parameter c. The model fit was assessed through residual analysis and graphical inspection of the observed versus predicted values. Curve fitting and visualization were performed in GraphPad Prism Version 10. This modeling approach follows established methodologies for analyzing toxicological dose–response data [50].

The nonlinear regression analysis method was chosen to determine toxicity, which involves fitting a mathematical (nonlinear) model to experimental data that describes the relationship between the dose of a toxic substance and the biological response. A 4-parameter log-logistic (4PL) nonlinear regression model was used to estimate the EC_50_ value.

### 3.4. Pigment Content

The chlorophyll a, chlorophyll b, and carotenoid contents were determined. Two mL of microalgal cell suspension were collected and centrifuged at 5000 rpm (4 °C, 10 min) (5804 R, Eppendorf, Wesseling-Berzdorf, Germany). Cells were resuspended in 2 mL of methanol (Chemsolute, Renningen, Germany) and shaken for 10 min (BenchMixerTM, Benchmark, Tempe, AZ, USA). The samples were placed in the refrigerator for 24 h at 4 °C and then centrifuged for 10 min at 5000 rpm. Chlorophyll a and b and carotenoids were determined in the supernatant using a UV-VIS spectrophotometer (Cary 300, Varian, Palo Alto, CA, USA) according to Gao et al. (2021) [51].

SOD and CAT activity, and MDA and ROS levels, were measured after 3 days and 7 days of exposure. For the SOD assay the supernatant was incubated with a reaction mixture consisting of 50 mM phosphate buffer, 0.1 mM EDTA (Chempur, Piekary Śląskie, Poland), 50 mM Na_2_CO_3_ (Chempur, Poland), 12 mM L-methionine (Fluka, Buchs, Switzerland), 50 µM NBT (AmBeed, Arlington Heights, IL, USA), and 10 µM ryboflavine (Sigma Aldrich, USA) in sunlight for 15 min. The spectrophotometer (Cary 300, Varian, Palo Alto, CA, USA) measurements were performed at a wavelength of 560 nm. One unit of SOD activity is defined as the amount of enzyme that causes a 50% inhibition of NBT [52]. The MDA assay was performed according to Das et al. (2022) [53]. Briefly, 2 mL of supernatants were collected and centrifuged (5000 rpm at 4 °C for 10 min). 2 mL of a thiobarbituric acid (TCI, Tokio, Japan) and trichloroacetic acid (Chempur, Poland) (0.25% and 10% *w*:*v*, respectively) mixture was added to the pellet, and the obtained mixture was kept at 95 °C for 30 min. Then, the mixture was quickly cooled and centrifuged. The supernatant measurements were conducted with a Cary UV-VIS spectrophotometer at a wavelength of 532 nm. A CAT assay was performed according to the protocol described in a previous report. The supernatant (100 μL) collected after centrifugation was treated with 2 mL of 10.8 mM H_2_O_2_ solution (Chempur, Poland) and 100 μL of 50 mM (pH 7 potassium phosphate buffer). The absorbance of the treated solution was measured at 240 nm using a UV-VIS spectrophotometer. The protein content was determined using the Bradford method [54]. ROS production was measured using 2′,7′-dichlorodihydrofluorescein diacetate (DCFH-DA) (Sigma Aldrich, St. Louis, MO, USA). Microalgae cells were separated from the growth medium by centrifugation at 5000 rpm for 10 min, and cells were treated with 100 nM DCFH-DA and incubated at 25 °C in the dark for 30 min. The fluorescence intensity of DCF was measured at an excitation wavelength of 485 nm and an emission wavelength of 530 nm using a spectrofluorometer (infinite M200, Tekan, Männedorf, Switzerland). The relative ROS level was calculated using Equation (3).

(3) $$ROS\;level=\frac{DCF\;FI\;treatment\;samples}{DCF\;FI\;control}\cdot 100\%$$

where *FI* is fluorescence intensity.

### 3.5. Morfological Properties

The morphology of *C. vulgaris* before and after its exposure to the GLY single and GLY-PE combined systems was observed using a scanning electron microscope (SEM) (VEGA 3 TESCAN, Brno, Czech Republic) in secondary electron (SE) mode. Analyses were performed using a beam voltage of 10 kV, a working distance of about 9.15 mm, and magnification of 5.00 kx. In order to improve the imaging process, an ultra-thin (5 nm) gold layer was sputtered on the sample surfaces. Then, 2 mL of cell suspensions were centrifuged at 4500 rpm for 10 min. The microalgal cells were then washed three times with 0.1 M PBS, centrifuged, embedded in agar, and fixed in 2.5% glutaraldehyde for 2 h at 4 °C. Subsequently, the microalga samples were dehydrated in an ethanol gradient (30%, 50%, 75%, 90%, 95%, and 100%).

### 3.6. Statistical Analysis

Quantitative data are expressed as means ± standard deviation (SD) from the indicated set of experiments. All statistical analyses were performed using GraphPad Prism (GraphPad Software Inc., La Jolla, CA, USA). The statistical values were calculated using an ANOVA test. Probability values below 0.05 (* *p* < 0.05) were considered to be statistically significant.

## 4. Conclusions

In this study, the influence of the GLY single and GLY-PE combined systems on the toxicity, pigment content, oxidative stress, and antioxidative enzymes in the freshwater microalgae *C. vulgaris* was thoroughly examined. The results confirmed that after 72 h of exposure of *C. vulgaris* to GLY and GLY-PE, the EC_50_ values were 9.77 mg/L and 2.31 mg/L, respectively. Our findings confirm that the presence of MPs can alter the toxicity of co-contaminants, often increasing their harmful effects. Moreover, their effect is alleviated after 7 days of exposure. A greater decrease in the chlorophyll a, chlorophyll b, and carotenoid content was observed after exposure to the GLY-PE combined system. The results of this study also showed that oxidative stress played a role in the mechanism of the toxic effect of the GLY single and GLY-PE combined systems on *C. vulgaris*. SOD and CAT activity, MDA content, and ROS production were evaluated. Higher ROS production was observed, and their accumulation was found to damage microalgal cells, as confirmed by a higher MDA rate for lipid peroxidation after 7 days of exposure. The GLY-PE combined system showed higher SOD and CAT activity when compared to the GLY single system.

In conclusion, the evidence obtained in this study suggests that the GLY-PE combined system has a significant effect the toxicity of pesticides and an influence on microalgal growth and physiology, highlighting potential ecological risks associated with its presence in aquatic environments. The limitation of this study is the use of only one type of PE-MP in a single size fraction (34–50 μm), which does not fully capture the diversity of MPs found in the environment. Furthermore, the concentrations used in the experiments were higher than typically reported in surface waters. However, this allowed us to determine EC_50_ values and capture clear toxic effects, which is a preliminary step towards research in more realistic conditions. Future tests involving different polymers and particle sizes, and lower environmental concentrations, are necessary to assess the true level of ecological risk.

More studies are needed to examine the combine effects of MPs and emerging contaminants on living organisms. The presented research in the freshwater microalgae *C. vulgaris* provides reference data for ecological risk assessments. The findings emphasize the importance of studying the interaction between MPs and pesticides in the aquatic environment.

## Data Availability

The raw data supporting the conclusions of this article will be made available by the authors on request.

## Abbreviations

The following abbreviations are used in this manuscript:

PE	polyethylene
GLY	glyphosate
PE-MPs	polyethylene microplastics
PS-MPs	polystyrene microplastics
PS-NH_2_	polystyrene cationic amino-modified nanoparticles
MDA	malondialdehyde
ROS	reactive oxygen species
SOD	superoxide dismutase
CAT	catalase
*C.*	*Chlorella*
SEM	Scanning Electron Microscopy
